# How Accurate Is Your Correlation? Different Methods Derive Different Results and Different Interpretations

**DOI:** 10.3389/fpsyg.2022.901412

**Published:** 2022-05-24

**Authors:** Kaiqi Shao, Majid Elahi Shirvan, Abdullah Alamer

**Affiliations:** ^1^Department of Foreign Languages, Hangzhou Dianzi University, Hangzhou, China; ^2^Department of Foreign Languages, University of Bojnord, Bojnord, Iran; ^3^Department of English, Imam Mohammad Ibn Saud Islamic University, Hofuf, Saudi Arabia; ^4^Department of English, King Faisal University, Al-Ahsa, Saudi Arabia

**Keywords:** correlation, quantitative methods, structural equation modeling (SEM), exploratory structural equation modeling (ESEM), confirmatory factor analysis (CFA), discriminant validity

## Abstract

Assessing the association between conceptual constructs are at the heart of quantitative research in educational and psychological research. Researchers apply different methods to the data to obtain results about the correlation between a set of variables. However, the question remains, how accurate are the results of the correlation obtained from these methods? Although various considerations should be taken to ensure accurate results, we focus on the types of analysis researchers apply to the data and discuss three methods most researchers use to obtain results about correlation. Particularly, we show how correlation results in bivariate correlation, confirmatory factor analysis (CFA), and exploratory structural equation modeling (ESEM) differ substantially in size. We observe that methods that assume independence of the items often generate inflated factor correlations whereas methods that relax this assumption present uninflated, thus more accurate correlations. Because factor correlations are inflated in bivariate correlation and CFA, the discriminant validity of the constructs is often unattainable. In these methods, the size of the correlation can be very large and biased. We discuss the reasons for this variation and suggest the type of correlation that researchers should select and report.

## Introduction

Understanding the association between theoretical constructs is at the heart of quantitative research. Researchers use correlation to understand how two or more variables are associated. Note that correlation does not infer causality especially when it is applied to cross-sectional data (Alamer and Lee, 2021). Beyond this, in first-generation analyses of correlations, which mainly involved bivariate correlation, the average or summary of the items’ score (or manifest score) is used to represent the given construct or dimension in the assessment. However, as noted by Marsh et al. (2009), the dimensionality assumption of the items belonging to only one factor leads to potential inflation in the magnitude of the correlation between variables. This limitation paved the way for the emergence of the second-generation methods of correlation based on structural equation modeling (SEM) such as confirmatory factor analysis (CFA) and exploratory structural equation modeling (ESEM). Researchers can obtain results of correlation between latent variables in CFA and ESEM, but empirical studies have highlighted significant differences between the two methods which we explain in this study. In this study, we present empirical evidence that different methods can generate distinct results of correlation, which eventually might change the interpretation of the results.

## Literature Review

### Measuring the Correlation Between Variables

#### First-Generation Methods

The relationship between variables is usually obtained by assessing how measures/scales that represent the variables are correlated. Analysts rarely use single items to represent a complex phenomenon because single items cannot appropriately capture the complexity inherent in theoretical concepts (Dörnyei and Taguchi, 2009). Researchers utilize measurement scales to get details about the constructs under investigation. Typically, few worded items (usually from three to ten items) targeting a particular concept are used. In first-generation analyses (such as bivariate correlation, regression, and *t*-test), these items are combined by averaging or summating their scores. This process is needed for such methods because it allows analysts to use one overall score that represents the construct in the analysis. Researchers, then, repeat this process for all subscales involved in the assessment. Obtaining total scores (manifest scores) of the items allows quantitative researchers to use correlation analysis (among other first-generation analyses). Nonetheless, Marsh et al. (2009) explain that manifest scores are derived from the assumption that items only reflect a single construct; thus, this assumption potentially inflates substantially the sizes of correlations between the variables (more to say about the relationship between the items and their factor in the subsequent section). Drawing on the same issue, Haenlein and Kaplan (2004) described the limitations of using first-generation techniques to examine correlation as they (i) postulate a simple model structure, (ii) require all variables to be observable (alternatively they are obtained by means of averaged or summed up the scores), and (iii) assume all variables are measured without measurement errors. These issues have an unavoidable impact on the quality of the results of correlation (among other analyses).

#### Second-Generation Methods

Beyond bivariate correlation, researchers have started to endorse second-generation methods (Hair et al., 2022) that are built on the property of structural equation modeling (SEM) to assess the associations between variables. Among these methods are confirmatory factor analysis (CFA) and exploratory structural equation modeling (ESEM) [see Alamer and Marsh (2022) and Alamer (2022b) for details and applied examples about ESEM]. CFA is a method that is used to understand the underlying factor structure of the constructs (Marsh et al., 2009; Morin et al., 2016). CFA gained more popularity in the field of SLA in last few decades as it uses the advantages of SEM, a key feature that exploratory factor analysis (EFA) is missing. Because it builds on SEM functionality, CFA is able to provide goodness-of-fit indices, examine competing model specifications, correlate items’ error terms (when theory and analysis support that), and assessment of between-group measurement invariance. In fact, the label “exploratory” only appears to be used for EFA after the invention of CFA (previously EFA was just called “factor analysis”) (Marsh et al., 2005). However, the label “exploratory” in EFA does not really imply that it should only be used for exploratory purposes; its statistical limitations are what prevented analysts from getting deeper results from EFA. For instance, in its basic form, EFA cannot generate the goodness-of-fit indices, be used in a predictive model, and be tested for invariance across different groups of participants.

One key feature of CFA is that items load *only* on the factors they are hypothesized to load on. Thus, cross-loadings across other untargeted factors are not allowed in CFA and are constrained to be *zero*. Early literature in EFA and CFA (Jöreskog, 1973) made the assumption that factors should be anchored in distinctive clusters of observed variables to constitute the latent variable. Nevertheless, this restrictive system of the measurement model has been challenged in the last decade (Marsh et al., 2009; Guay et al., 2015; Morin et al., 2016, 2020; Alamer, 2021a,2022b; Alamer and Marsh, 2022). This is because conceptual constructs have certain levels of similarities; they can overlap especially when they are conceptually related. Consider, for example, the measurement model reported in Alamer and Marsh (2022) study where two constructs, *harmonious passion* and *obsessive passion*, were involved in the analysis. To provide context to the example, harmonious passion reflects the strong desire to freely engage in language activity whereas obsessive passion reflects the controlled pressure combined with an uncontrollable urge to partake in the language activity. In the L2-Passion scale (Alamer and Marsh, 2022), an item in harmonious passion reads “the new things that I discover in English allow me to appreciate it even more” while an item in obsessive passion reads “learning English is the only thing that really turns me on.” One can see how these two items belong to two different types of passion, but also each item seems to present significant true scores on the other (untargeted) type of passion. If the item on harmonious passion has no role at all to play in contributing to the meaning of the other factor, then why factor correlation is relatively high? such inflated factor correlation may be the result of the overly restrictive independent cluster representation of CFA.

With these observations in mind, why do analysts still prefer CFA even though it often produces unacceptable results both in the fit indices and factor correlation? Marsh et al. (2009) provide an answer to this question as they explain that “because of the recent dominance of CFA approaches to factor analysis, applied researchers have persisted with dubious approaches to CFA in the mistaken believe that EFA approaches were no longer acceptable. These misconceptions have been reinforced by the erroneous beliefs that many of the methodological advances associated with CFAs… are not possible when latent constructs are inferred on the basis of EFAs rather than CFAs” (p. 441).

Alternatively, research shows that certain levels of true scores can be relevant for other conceptually related constructs (Guay et al., 2015; Morin et al., 2016, 2020). This observation was confirmed by Alamer and Marsh (2022) as they found CFA fit indices were not acceptable for the L2-Passion scale. The researchers noted that CFA was rather a restrictive structure in that it ignores cross-loadings among the items of two conceptually related constructs, which, in turn, resulted in inflated factor correlation. To solve this, the researchers applied the recently developed method, ESEM (explained next). What they found is that ESEM better fits the measurement model and provided uninflated factor correlation.

### Exploratory Structural Equation Modeling as an Alternative Method of Correlation

**FIGURE 1:**
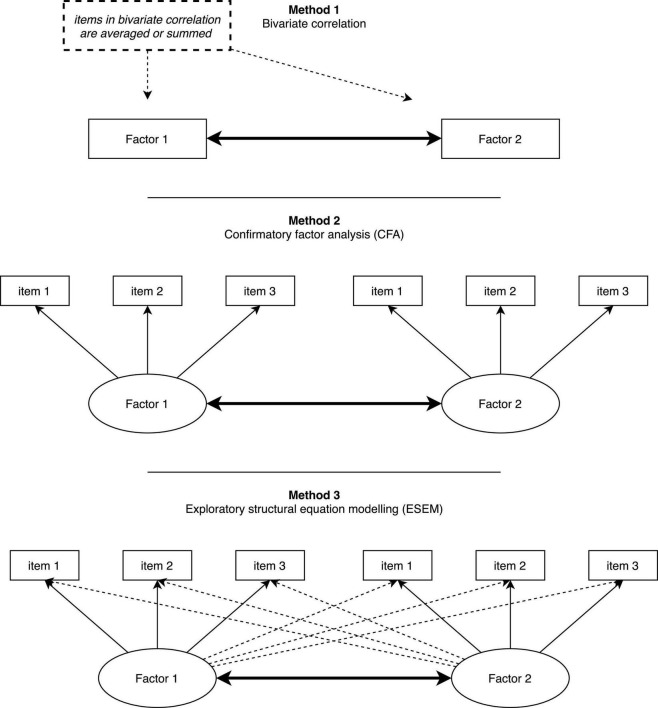
Visual representation of the correlation in bivariate correlation, CFA, and ESEM.

So, why does ESEM outperform CFA in empirical studies? In essence, ESEM shares a fundamental property with EFA in that both methods allow items to cross-load. However, they differ in that ESEM builds directly on SEM property (same as CFA). Hence, all SEM features used in the CFA have been successfully transferred (or brought back) to the EFA. As research has shown (Marsh et al., 2009; Morin et al., 2020; Alamer, 2021b), conceptual as well as empirical evidence is in favor of allowing cross-loadings to be estimated, particularly when conceptually related constructs are involved in the measurement model. When cross-loadings are allowed to be estimated, factor correlation appears to be unbiased (even when cross-loadings are very small), and model fit indices improve substantially (Marsh et al., 2020). Accordingly, and most importantly, the correlation obtained from ESEM is deemed more realistic and reflects a more accurate correlation magnitude in the population (Alamer, 2022b). We show to the readers an applied example of correlation generated from bivariate correlation, CFA, and ESEM. Figure 1 represents visually the differences in correlation between the three methods.

### Example From Real Data Using Self-Determination Theory in Second Language Scale

As a macro theory of motivation, self-determination theory (SDT) has been used in several life domains to examine what motivates individuals to follow their goals (Deci and Ryan, 2000). The theory contends the existence of two general types of motivation, autonomous motivation and controlled motivation with each having two sub-types of regulations. Autonomous motivation refers to the quality of individuals’ motivation being volitional. The first regulation under autonomous motivation is intrinsic regulation which represents language learners’ inherent inclination toward carrying out the language tasks. Identified regulation refers to the value and importance language learners attach while doing language tasks (Alamer and Lee, 2019; Alamer and Al Khateeb, 2021). On the other hand, two regulations “introjected regulation” and “external regulation” constitute the overarching construct of controlled motivation (Ryan and Deci, 2020). Introjected regulation refers to the inner and outer pressure that individuals experience to undertake learning activities. External regulation reflects the desire to learn and engage in language activity because of tangible and intangible rewards or the avoidance of punishment (Alamer and Almulhim, 2021).

To make our discussion about correlation concise, the example in this methodological paper reports only on the correlation between two variables, intrinsic regulation and identified regulation under the global construct “autonomous motivation.” We draw on the empirical results reported in the study of Alamer (2022a) which tested the construct validity of the self-determination theory in second language (SDT-L2) scale (readers are referred to that study for more details about the data). The author assessed the factorial structure of the constructs *via* ESEM. He found support for the bifactor ESEM model over bifactor CFA in goodness-of-fit indices and meaningful factor loadings in both the specific and general factors. Among the four constructs of SDT-L2 scale, two constructs, intrinsic regulation and identified regulation are explained in the present study (see the Appendix for scale items). Each construct has 5 items (collectively comprising 10 items) that are based on a 5-point Likert-type response format. The bivariate correlation between the variables reported in that study was *r* = 0.69, *p* < 0.001. Alamer (2022a) study did not include the standard CFA and ESEM but only the bifactor solutions, thus we extend that by reporting the correlation of standard CFA and ESEM using the same dataset (readers are referred to that study for more details about the descriptive statistics and the sample). After running the analysis through Mplus 8.1, we found that CFA and ESEM have resulted in a distinct size of correlation (CFA *r* = 0.82, *p* < 0.001; ESEM *r* = 0.51, *p* < 0.001). Table 1 describes the differences between the three methods. Although the fit indices are not the focus of our discussion we report them accordingly: (CFA: χ^2^ = 116.93, *df* = 34, *p* < 0.001, SRMR = 0.05, RMSEA = 0.11, RMSEA Low and Hi 95% CI [0.09, 0.13], CFI = 0.92, TLI = 0.90; ESEM: χ^2^ = 81.72, *df* = 26, *p* < 0.001, SRMR = 0.03, RMSEA = 0.09, RMSEA Low and Hi 95% CI [0.09, 0.12], CFI = 0.95, TLI = 0.92).

**TABLE 1:** Factor correlations obtained from bivariate correlation, CFA, and ESEM.

	Bivariate correlation	CFA	ESEM
	0.69	0.82	0.51
Deviation from ESEM	Δ*r* = +0.18	Δ*r* = +0.31	−

*All correlations are significant at p < 0.001.*

The reduced factor correlation between the two variables in the ESEM can be said to reflect a more realistic, thus more precise, representation of the association between intrinsic regulation and identified regulation. This is because, as noted by Morin et al. (2016, 2020), certain levels of true scores of items on the non-target factors should be expected and accepted in ESEM solutions. If we use the L2 guidelines to interpret our correlations (i.e., Plonsky and Oswald, 2014), we will conclude that bivariate correlation, and particularly the CFA, have resulted in correlations that are large in size (very large in CFA) while the correlation in ESEM has been reduced significantly to a medium effect size. The small differences in CFA and bivariate correlation can be attributed to the fact that bivariate correlation aggregates the items of the factor into one sum or averaged score; thus, results are not likely to be identical. Hence, it can be clear that different analyses result in different magnitudes of correlation, and with different magnitudes come distinct interpretations of the results. The weaker correlation of the two regulations in ESEM represents an uninflated and unbiased result due to the cross-loading of their items. More specifically, despite the fact that the two variables refer to different types of motivational regulation, they provide significant true scores on each other because both tap on and relate to the general construct “autonomous motivation” (Deci and Ryan, 2000; Alamer, 2021a). The cross-loadings of intrinsic regulation items on identified regulation can be supported by the fact that items on intrinsic regulation contributed, albeit weakly, to the meaning of the construct of identified regulation, and vice versa. For example, an item on intrinsic regulation reads “for the satisfaction I feel when I speak and write in English” cross-loaded on identified regulation [0.14, *p* > 0.05 as reported in Alamer (2021a)]. This cross-loading, albeit weak, can be said meaningful because certain levels of learning satisfaction can be also associated with self-growth and personal value as expressed in identified regulation (readers are referred to the original report for a fuller discussion of the cross-loadings). With such a flexible system, factor correlation reduces to a more realistic level. Thus, it can be noticeable that ESEM relaxes the strong assumption of the independent clusters model of CFA which assumes all items have zero factor loadings on all untargeted factors other than the one they are hypothesized to relate to Marsh et al. (2020). Consequently, fit indices in ESEM improve substantially compared with CFA. Such improvement may indicate that factor correlation (among other results) in ESEM better represent the data as well.

### Effects of Estimation and Rotation Methods

We want to highlight that using different rotation methods in ESEM may result in slightly different loadings, which might lead to different sizes of correlations. However, two mostly used rotations “Target rotation” and “Oblimin rotation,” are recommended depending on the nature of the investigation [see Morin et al. (2020) for greater a discussion], and their correlation results are often comparable. Another area that needs to be considered is the estimator used in ESEM. The method selected plays a role in estimating the path coefficients and factor correlations. The most common method used to estimate the model is maximum likelihood (ML). But robust ML (MLR) is better suited when data does not fully satisfy normality assumption. Apart from ML and MLR, some estimators make no distributional assumptions about the observed variables and, thus better suit ordinal data such as diagonally weighted least squares (WLSMV, also called DWLS). Simulation studies noted that using WLSMV results in inflated factor correlation compared to MLR when the sample size is modest N < 200 and the data is relatively non-normal [see Li (2016) for a greater discussion]. It is recommended that researchers use MLR when the normality is not substantially violated and that the scale has 5 or more categories (which is commonly used in Likert scale questionnaires), while WLSMV estimator is justified when the scale has 4 categories or less (Shi and Maydeu-Olivares, 2020).

We also note that ESEM has a specification that assumes the co-existence of a global factor called, bifactor ESEM. In bifactor ESEM, specific factors and general factors were specified as orthogonal (Morin et al., 2016; Alamer, 2021b). That is, this type of model requires that correlations between all factors be constrained to zero [see Alamer (2021a) for an application of bifactor models]. Therefore, when the researchers’ goal is to evaluate factor correlations, they should first consider standard ESEM to obtain results about correlation. Then, they can pursue the analysis and use bifactor models (if theory suggests that).

## Summary and Recommendations

In this methodological paper, we have discussed three types of approaches that researchers mostly apply to obtain results about correlation. Correlation is one of the most widely used quantitative analyses that researchers use to understand how L2 variables are interrelated. Beyond the layman’s belief that the significance test (i.e., whether the *p*-value is less than 0.05) is the ultimate objective of correlation (Plonsky and Oswald, 2014; Alamer and Lee, 2021), researchers need to select an approach that represents reality in the population as close as possible. Our results with data from self-determination theory in second language (SDT-L2) scale, specifically the association between intrinsic regulation and identified regulation, show that analyses that assume independent item loadings (e.g., bivariate correlation and CFA) have provided biased factor correlation, thus negatively impacting the interpretation of the results. Reviewing correlation results other than those reported in the present study, one can find examples from SLA literature of factor correlations that reach *r* = 0.90 in CFA (see, for example, Park, 2011) and many other studies report correlation that ranges between *r* = 0.70 and *r* = 0.90. Statistically, *r* = 0.90 is a very large magnitude and implies that the two factors have 81% of shared variance (i.e., they are 81% similar), which empirically detracts from the discriminant validity of the factors. Arguably, it is not unmanageable to assume a distinct meaning of factors when they share such a substantial amount of variance. We suggest that factor correlation should not exceed 0.70 in the measurement model because exceeding this cutoff value indicate that the factors share more than 50% of similarities. When these two highly correlated factors are employed in a structural model, the solution is likely to face collinearity issues, which result in biased path coefficients.

This observation also applies to bivariate correlation, albeit at a decreased magnitude level. Conversely, when ESEM is employed, results of correlation would better support the discriminant validity of the factors and can be said to be more realistically represent the population. As such, instead of bivariate correlation or CFA, we suggest researchers apply ESEM to understand how latent variables are associated. Note that discriminant validity is not only achieved by factor correlation but also through the weak cross-loadings of the items on the untargeted factors [see Alamer and Marsh (2022) for greater details]. Further, not all ESEM solutions will result in significantly reduced factor correlation because it depends on the nature of the factors involved in the assessment. Nevertheless, ESEM often results in reduced factor correlation relative to CFA.

In addition, our review on correlation does not cover the full possibilities of gaining correlation between the factors; we, instead, have discussed the most widely used methods in the field and commented on them. For example, researchers may obtain correlation from partial least squares SEM (PLS-SEM) [Hair et al., 2019; see also Henseler (2020)] and the results are likely to be different because the estimation method is different. We also want to highlight that we did not comment on the type of the scale (measurement scales) or the normality of the data as each type holds particular consideration in the analysis. We instead focused on variables that are perceived as continuous or at least treated as interval such as the case with the symmetric Likert scale (Hair et al., 2019). At the time of this publication, only Mplus and R can run ESEM and we hope researchers would endorse this method in their research. A recent study that introduces ESEM to SLA research that included applied examples, the syntax required for Mplus with data that is publicly available is currently published (i.e., Alamer and Marsh, 2022). Among the wealth of its benefits, we think that ESEM can be an alternative analytical tool to understand precisely how constructs/measures are correlated. It would be more accurate for the L2 quantitative researchers to endorse ESEM for future empirical studies to investigate the association between the variables.

## Data Availability Statement

The raw data supporting the conclusions of this article will be made available by the authors, without undue reservation. Requests to access the datasets should be directed to AA, alamer.aaa@gmail.com.

## Ethics Statement

The studies involving human participants were reviewed and approved by Imam Mohammad Ibn Saud Islamic University (IMSIU). The patients/participants provided their written informed consent to participate in this study.

## Author Contributions

AA was responsible for the research design, data collection, and draft writing of this study. ME helped with the theory, literature review, and arrangement of the article. KS helped with the theory, review, and revision of the article. All authors contributed to the article and approved the submitted version.

## Conflict of Interest

The authors declare that the research was conducted in the absence of any commercial or financial relationships that could be construed as a potential conflict of interest.

## Publisher’s Note

All claims expressed in this article are solely those of the authors and do not necessarily represent those of their affiliated organizations, or those of the publisher, the editors and the reviewers. Any product that may be evaluated in this article, or claim that may be made by its manufacturer, is not guaranteed or endorsed by the publisher.

## Appendix

### Self-Determination Theory of Second Language Subscale (SDT-L2)*

**Table:** Why are you learning English?

Item
**Autonomous motivation**
***Intrinsic orientation***
Because I enjoy learning English
Because of the pleasure I get when hear and read English
For the satisfaction I feel when I speak and write in English
For the enjoyment I experience when I achieve a new goal in English learning
Because learning English is a fun activity in and of itself
***Identified orientation***
Because learning English is important for my personal growth
Because learning English can open new opportunities and possibilities for me
For the value it holds in my self-development
Because learning English is important for my current and future studies
Because learning English allows me to read and hear English-based materials that are necessary for my personal success

**Taken from Alamer (2022a).*
